# Vital imaging of H9c2 myoblasts exposed to *tert*-butylhydroperoxide – characterization of morphological features of cell death

**DOI:** 10.1186/1471-2121-8-11

**Published:** 2007-03-16

**Authors:** Vilma A Sardão, Paulo J Oliveira, Jon Holy, Catarina R Oliveira, Kendall B Wallace

**Affiliations:** 1 Center for Neurosciences and Cellular Biology, University of Coimbra, Coimbra, Portugal; 2 Department of Anatomy, Microbiology and Pathology, University of Minnesota-Medical School, Duluth, USA; 3 Department of Biochemistry and Molecular Biology, University of Minnesota-Medical Medical School, Duluth, USA

## Abstract

**Background:**

When exposed to oxidative conditions, cells suffer not only biochemical alterations, but also morphologic changes. Oxidative stress is a condition induced by some pro-oxidant compounds, such as by tert-butylhydroperoxide (tBHP) and can also be induced *in vivo *by ischemia/reperfusion conditions, which is very common in cardiac tissue. The cell line H9c2 has been used as an *in vitro *cellular model for both skeletal and cardiac muscle. Understanding how these cells respond to oxidative agents may furnish novel insights into how cardiac and skeletal tissues respond to oxidative stress conditions. The objective of this work was to characterize, through vital imaging, morphological alterations and the appearance of apoptotic hallmarks, with a special focus on mitochondrial changes, upon exposure of H9c2 cells to tBHP.

**Results:**

When exposed to tBHP, an increase in intracellular oxidative stress was detected in H9c2 cells by epifluorescence microscopy, which was accompanied by an increase in cell death that was prevented by the antioxidants Trolox and N-acetylcysteine. Several morphological alterations characteristic of apoptosis were noted, including changes in nuclear morphology, translocation of phosphatidylserine to the outer leaflet of the cell membrane, and cell blebbing. An increase in the exposure period or in tBHP concentration resulted in a clear loss of membrane integrity, which is characteristic of necrosis. Changes in mitochondrial morphology, consisting of a transition from long filaments to small and round fragments, were also detected in H9c2 cells after treatment with tBHP. Bax aggregates near mitochondrial networks were formed after short periods of incubation.

**Conclusion:**

Vital imaging of alterations in cell morphology is a useful method to characterize cellular responses to oxidative stress. In the present work, we report two distinct patterns of morphological alterations in H9c2 cells exposed to tBHP, a pro-oxidant agent frequently used as model to induce oxidative stress. In particular, dynamic changes in mitochondrial networks could be visualized, which appear to be centrally involved in how these cells respond to oxidative stress. The data also indicate that the cause of H9c2 cell death following tBHP exposure is increased intracellular oxidative stress.

## Background

The myoblast cell line H9c2, derived from embryonic rat heart [1], has been used as an *in vitro *model for both skeletal and cardiac muscle. H9c2 cells show electrophysiological and biochemical properties of both skeletal and cardiac tissues, including depolarization in response to acetylcholine [1], and rapid activation of calcium currents through L-Type channels [2-4]. An interesting feature of this cell line is its ability to differentiate from mono-nucleated myoblasts to myotubes upon reduction of serum concentration [2]. Accompanying myotube formation is the expression of myogenic transcription factors [5], calcium channel proteins [6], and the LIM protein FHL2 [7]. During the differentiation process, cells retain several elements of the electrical and hormonal signaling pathway of cardiac cells [1] and have therefore become an accepted *in vitro *model to study the effects of ischemia and diabetes on the heart [8-10].

L'Ecuyer et al. (2001) [11] demonstrated that H9c2 cells can be used to study free radical production, and can be engineered to express foreign genes at controllable levels, making them a suitable system to study molecular responses to oxidative damage. Previous works in this area include studies of oxidative cell damage caused by doxorubicin [11-13], ischemia and reperfusion [14], hydrogen peroxide [15,16] and peroxynitrite [17].

*Tert*-butyl hydroperoxide (tBHP) is one of the most common pro-oxidant agents used to evaluate the effects of oxidative stress [18], and it has been found to induce cell death in several cell lines such as U937 [19], HepG2 [20], and primary cultures of cardiomyocytes [21] and hepatocytes [18,22]. According to the cell line used, induction of cell death by tBHP is characterized by cytochrome c release [23], increased expression of p53 [24], induction of the mitochondrial permeability transition pore [25], TUNEL-positive labeling, phosphatidylserine (PS) exposure and caspase 3 activation [26].

However, little is currently known regarding the specific behavioral responses of H9c2 cells exposed to pro-oxidants. Therefore, the objective of the present work was to use tBHP to study the morphological events accompanying pro-oxidant damage in H9c2 myoblasts, with a special focus on mitochondrial changes. Furthermore, the morphological alterations resulting from tBHP treatment were correlated with well established markers of apoptosis and necrosis, including chromatin condensation, PS exposure in the outer leaflet of the cell membrane, BAX translocation, and loss of plasma membrane integrity. This study provides novel information regarding the behavioral responses of a muscle cell line to pro-oxidant damage. Furthermore, the data obtained permits the extension of our understanding of the conditions that drive cells from apoptosis to necrosis, which are distinct methods of cell death that embody dissimilar medical consequences for the affected target organ.

## Results

**Figure 1:**
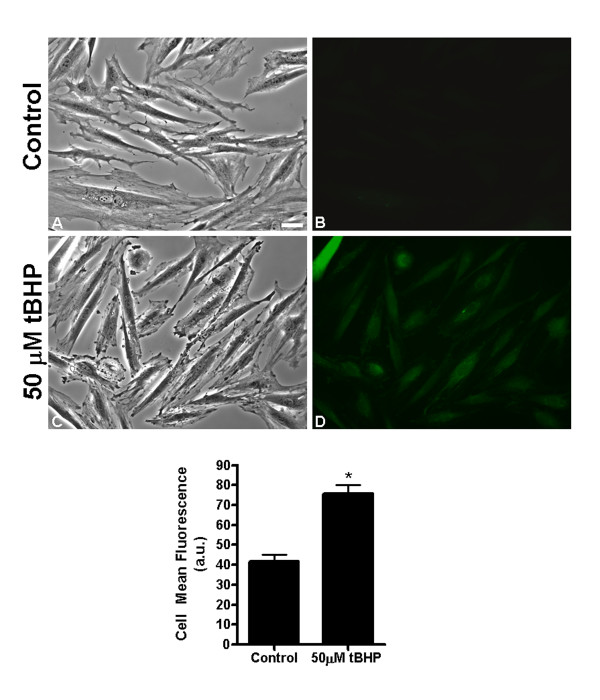
**tBHP induced and increase intracellular oxidative stress in H9c2 cells**. (Top panel) H9c2 cells were exposed to 0 (panel A-B) or 50 μM tBHP (panel C-D) for 1 hour. Increase in intracellular oxidative stress was detected by oxidation of dichlorofluorescein. Fluorescence observed in panel B and D is proportional to the degree of oxidation. The corresponding DIC images can be observed in panels A and C. Images are representative from 3 different cell preparations. A-D are the same magnification; scale bar in A = 20 μm. (Bottom panel) Quantification of cell DCF fluorescence intensity. Four different fields were analyzed in each experiment. Data represents the mean ± SEM of 3 different cell preparations. *p < 0.05 vs control.

Before analyzing changes in cellular morphology induced by tBHP, the pro-oxidant action of this compound was confirmed in H9c2 cells. Increased intracellular oxidative stress was detected by the oxidation of dichlorofluorescein (DCF) in cells that were exposed to 50 μM tBHP for one hour (Figure 1). Most cells suffering from high oxidative stress remained well-spread (compare Fig. 1A and 1C); however, small membrane blebs started to become evident at the cell periphery. Quantitation of mean DCF fluorescence intensity in control and treated cells was also performed (Figure 1 – bottom panel). The results showed a statistically significant increase in DCF oxidation, confirming increased intracellular oxidative stress after 1 hour treatment with 50 μM tBHP.

**Figure 2:**
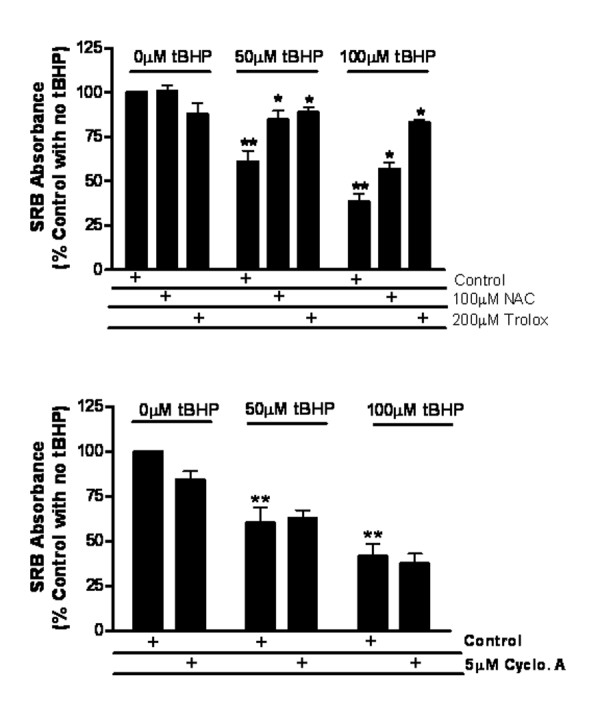
**Cytotoxicity of tBHP and the effects of NAC, Trolox, Cyclosporin-A was analyzed by sulforhodamine B dye-binding assay**. Effects of NAC and Trolox (top pannel) or cyclosporin-A (bottom panel) on tBHP-induced cell loss. Cell treatment and analysis were performed as described in the methods section. Data represent the mean ± SEM of 3 different cell preparations. *p < 0.05 vs respective control (tBHP without inhibitor); ** *p *< 0.05 vs control (no tBHP added).

By measuring cell numbers with the sulforhodamine B dye method [27], the protective effect of antioxidants N-acetylcysteine and Trolox (a water-soluble vitamin E derivative) was evaluated against tBHP cytotoxicity (Figure 2, top panel). The results showed a significant decrease in H9c2 cell number after 6 hours of treatment with 50 and 100 μM tBHP, compared with the control. The decrease in cell numbers due to tBHP induced cell death was prevented by N-acetylcysteine and Trolox (Figure 2, top). In order to analyze if the cytotoxic effect of tBHP was related to the induction of the mitochondrial permeability transition (MPT), an oxidative stress-dependent phenomenon characterized by the loss of the impermeability of the mitochondrial inner membrane, we also analyzed the effect of cyclosporin-A (a well-known MPT inhibitor). As presented in Figure 2, bottom panel, cyclosporin-A did not prevent the toxicity of tBHP.

**Figure 3:**
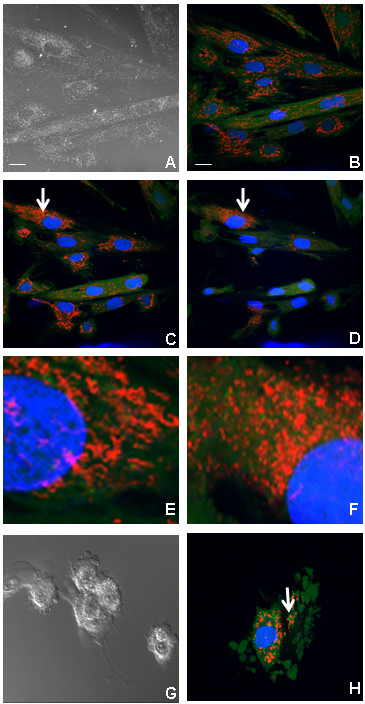
**Alterations in cellular and mitochondrial morphology induced by tBHP**. (A) DIC images from untreated cells, showing normal membrane morphology. (B) Laser scanning confocal microscopy image from untreated cells showing triple labeling with Hoechst 33342 (blue), calcein (green) and TMRM (red). (C, D) Laser scanning confocal microscopy showing the fluorescence of the three dyes followed with the time, (C) 45 min, and (D) 90 minutes, after treatment with 80 μM tBHP. (E) Magnification of the cell indicated by an arrow in panel C and (F) magnification of the cell indicated by an arrow in panel D. (G) DIC images from H9c2 cells treated with 25 μM tBHP for three hours. (H) Laser scanning confocal microscopy (LSCM) image from cells treated with 25 μM tBHP during three hours. Images are representative from 3 different cell preparations. Panels B-D and G, H are the same magnification. Scale bar corresponds to 20 μm.

Morphological alterations of H9C2 cells after treatment with tBHP were analyzed by epifluorescence microscopy (Fig. 3). Control (untreated) cells are well spread, strongly labeled by calcein, and exhibit filamentous mitochondria distributed throughout the cytoplasm (Fig. 3B). However, after tBHP treatment, mitochondria lost their filamentous shape and displayed several morphological alterations. After 45 min exposure to 80 μM tBHP, mitochondria were concentrated close to the nuclear region and in some cells, the amount of polarized mitochondria (mitochondria with negative-inside electric potential) decreased (Fig. 3C). At 90 minutes, the mitochondrial network in some cells showed signs of degradation, being converted from a filament-like form to small round mitochondria (Fig. 3D and 3F). Exposure of H9c2 cells to 25 μM tBHP for three hours induced some cells to round up and exhibit increased membrane blebbing (Fig. 3G). Morphologically, cells treated with 25 μM tBHP display peripheral membrane blebbing, followed by rounding up of cells as membrane blebbing continues.

Interestingly, examination of triple-labeled cells (Hoechst, calcein and TMRM) by confocal microscopy, demonstrated that mitochondria could remain polarized inside apoptotic bodies (Fig 3H, arrow).

**Figure 4:**
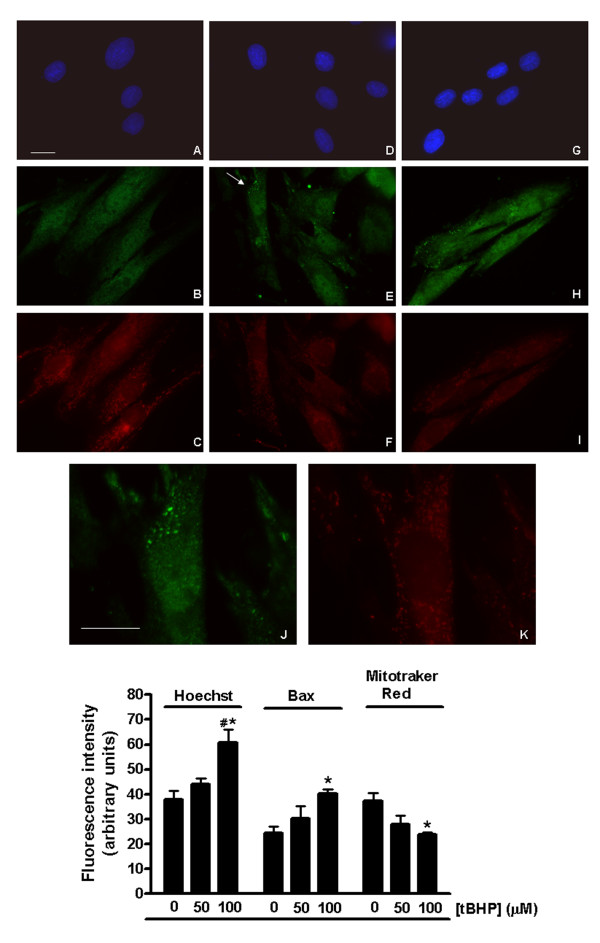
**tBHP induces formation of BAX clusters close to the mitochondrial network**. (Top panel) Control and tBHP treated cells were incubated with 125 nM Mitotracker red (red) for 30 min previous to fixation and subsequently labeled with an antibody to BAX (green) and counterstained with Hoechst (blue). (A-C) Control cells showing a homogeneous BAX distribution throughout the cell and filament-shaped mitochondria. (D-F) and (G-I) H9c2 cells treated with 50 μM and 100 μM tBHP, respectively, during 1 hour, showing BAX aggregates located close to the mitochondrial network and a change in mitochondria morphology from a filament-like form to small round mitochondria. (J) and (K) Magnification of the cell indicated by an arrow in Panel E (J-BAX immunolabeling; K-Mitotracker red labeling). Images are representative from 3 different cell preparations. Scale bar corresponds to 20 μM. (Bottom panel) Quantification of the cell fluorescence intensity of Hoechst, Bax immunolabeling and Mitotracker red. Data represent the mean ± SEM of 3 different cell preparations. *p < 0.05 vs 0 μM tBHP, ^# ^*p *< 0.05 vs 50 μM tBHP.

To verify if the morphological changes resulting from tBHP treatment were accompanied by changes in mitochondrial-associated apoptotic proteins, H9c2 cells were fixed and labeled with an antibody to the pro-apoptotic protein Bax. After 1 hour treatment with 50 μM tBHP, we observed the formation of Bax aggregates close to the mitochondrial network (figure 4, panel E and J). Bax clusters were also observed in some cells after treatment with 100 μM tBHP (figure 4, panel H). Examination of cells at high magnification indicates that the Bax aggregates are located both in and around clusters of mitochondria (figure 4, panel J and K). Statistical analyses of cell fluorescence intensities for Hoechst, Bax immunolabeling and Mitotracker Red (Figure 4, bottom panel) were also performed. The results show a statistically significant increase for both Hoechst and Bax fluorescence after treatment with 100 μM tBHP, as opposed to a decrease in the fluorescence intensity of Mitotracker Red. Bax clusters located in and around mitochondria were also observed after treatment with 50 μM tBHP.

**Figure 5:**
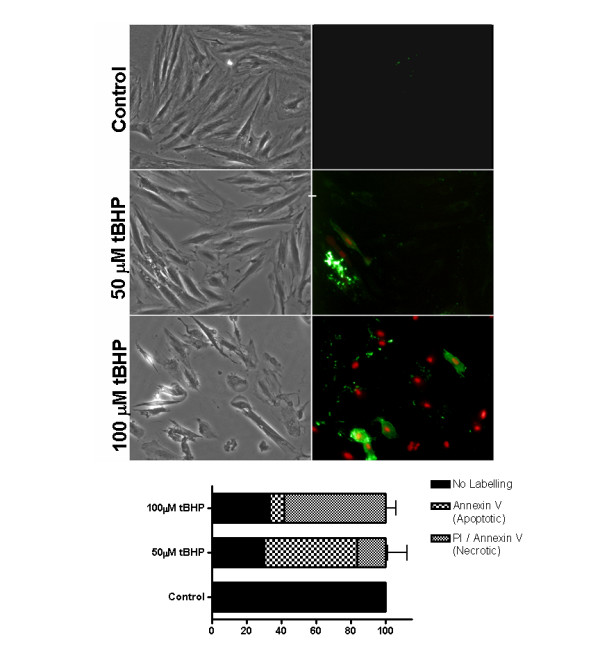
**tBHP induces the translocation of phosphatidylserine to the outer leaflet of the cellular membrane**. (Top panel) After treatment with 0, 50 and 100 μM tBHP, for 1 hour, H9c2 cell were labeled with Annexin V and propidium iodide. Left panels: DIC images of H9c2 cells, treated with 0, 50 and 100 μM tBHP. Right panels: corresponding epifluorescence microscopy images of propidium iodide (red) and Annexin V (green). Images are representative from 3 different experiments. All images are the same magnification; scale bar = 20 μm. (Bottom panel) Statistical analysis of the population of control cells or cells treated with increasing concentrations of tBHP, showing the relation between treatment and annexin V/propidium iodide labeling. Analysis was performed by counting cells without labeling (healthy cells), annexin V labeling (apoptotic cells) and PI/Annexin V double labeling (necrotic cells). Data represent the mean ± SEM of 3 different cell preparations.

To examine whether phosphatidylserine (PS) exposure occurs in H9c2 cells treated with tBHP, control and oxidant-treated cultures were dual-labeled with annexin V and propidium iodide (PI). Because PI is excluded from cells with intact plasma membranes, it was used to determine whether annexin V labeling was due to externalization of PS, or instead resulted from a loss of membrane integrity (Fig. 5). Control cells were neither PI nor Annexin V positive, while treatment with 50 μM tBHP for 1 hour caused some cells to become Annexin V positive (0 vs. 53.3 ± 17.6%, p < 0.05 vs control). Although an increase in double PI/Annexin V labeling (necrotic cells) was observed (0 vs. 16.7 ± 12.0%), the result was not statistically significant (p > 0.05 vs control). In contrast, one hour treatment with 100 μM tBHP caused a significant increase in the labeling of both PI and Annexin V (0 vs. 58.4 ± 5.7%, p < 0.05 vs control). No differences in Annexin V labeling were found between the 100 μM tBHP treatment groups and controls (0 vs. 8.3 ± 8.3%, p > 0.05 vs control). The results demonstrated that 100 μM tBHP could lead to both necrosis and accelerated apoptosis after just 1 hour of treatment.

**Figure 6:**
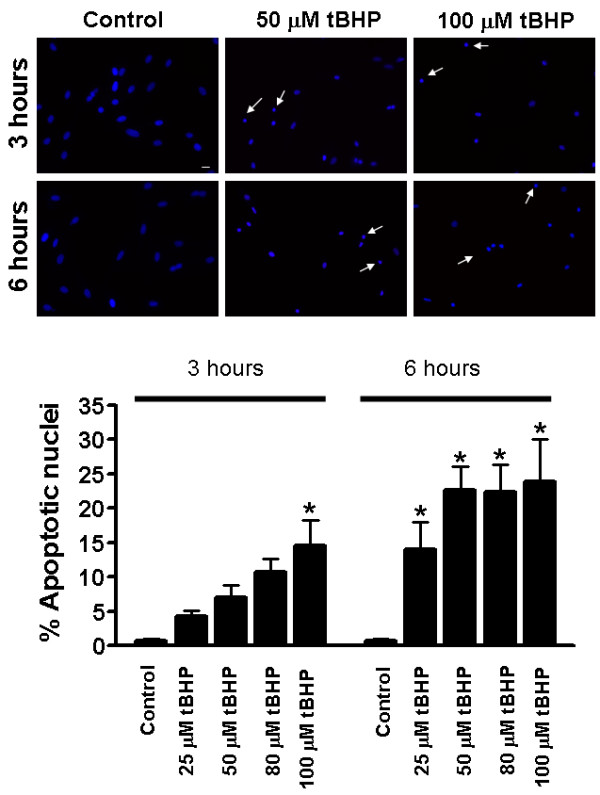
**Quantitation of apoptotic cells by Hoechst labeling methods**. (Top panel) Epifluorescence microscopy images of nuclei showing H9c2 cells treated with 0, 50 and 100 μM tBHP during 3 and 6 hours. Changes in nuclear morphology characteristic of apoptosis (arrows) were detected in Hoechst 33342-stained cells. Scale bar = 20 μm. (Bottom panel) The numbers of apoptotic nuclei were counted and expressed as the percentage of total cells counted (approximately 200 cells per coverslip). Data represents the mean ± SEM of 5 different experiments (bars indicate standard error). *p < 0.05 vs control.

Nuclear morphologic changes typical of apoptosis were also detected by epifluorescence observation of tBHP-treated cells stained with Hoechst 33342 (Fig. 6, upper panel). After treatment with different concentrations of tBHP (25–100 μM) during 3 hours, we detected a dose-dependent increase in the number of nuclei showing condensed chromatin. However, a statistically significant difference was only found for 100 μM tBHP (Fig. 6, lower panel). On the other hand, 6 hours of tBHP exposure induced a statistically significant increase in the number of apoptotic nuclei for all concentrations tested (Fig 6, lower panel). No differences between control and treated cells were observed after 1 hour of treatment (data not shown).

**Figure 7:**
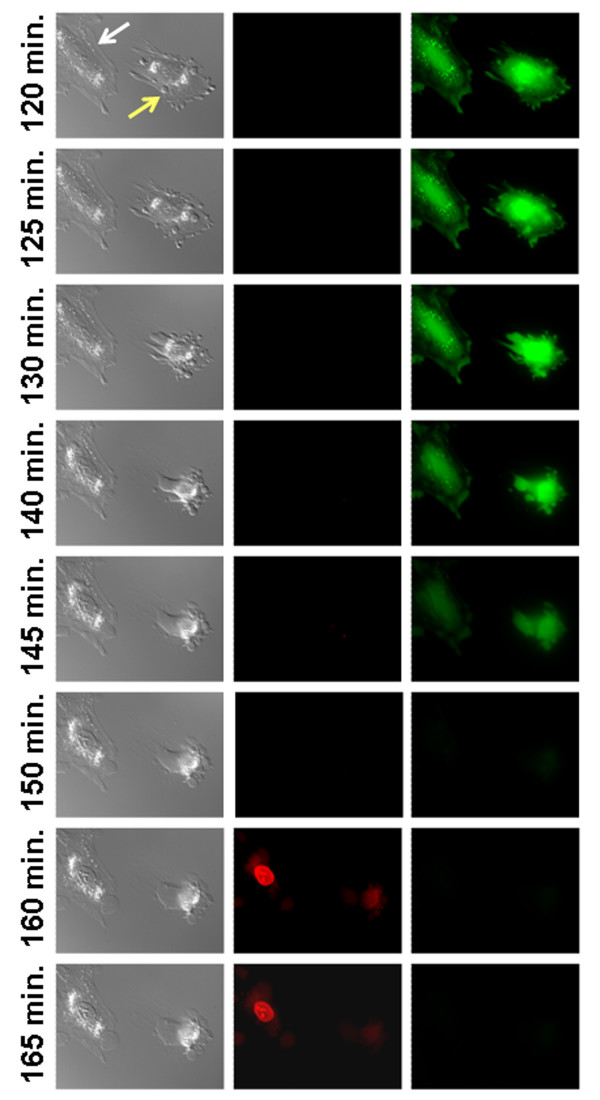
**Real-time epifluorescence microscopy of calcein and ethidium homodimer fluorescence after treatment with 100 μM tBHP**. DIC (left panels), calcein (right panels) and EH-1(middle panels) epifluorescence microscopy images, collected every two minutes after treatment with 100 μM of tBHP. Images are representative of still micrographs obtained from the time-lapse of cells treated with tBHP and labeled with calcein-AM and EH-1.

Finally, we were interested in following by real-time epifluorescence microscopy the transition between apoptosis and necrosis after treatment with 100 μM tBHP. In order to analyze the transition, the loss of calcein fluorescence and an increase in nuclear-derived ethidium homodimer fluorescence were detected after treatment with tBHP. Figure 7 shows sequential images, beginning at 120 min. of tBHP exposure, from one field of view that contained one blebbing cell and one well-spread cell. The well-spread cell (white arrow) slightly shrank in size without significant membrane blebbing and suffered necrosis, as observed by the loss of calcein fluorescence, increase in ethidium homodimer red fluorescence and visible extrusion of cytosolic contents. The blebbing cell (yellow arrow) shrank in size, but ultimately also underwent necrosis, as observed by the loss of calcein fluorescence, gain in ethidium homodimer red fluorescence and visible extrusion of cytosolic contents. Notably, the well-spread cell maintained the majority of its basic structure unaltered while the blebbing cell lost its original form and rounded up. Strikingly, at 150 min., no fluorescence whatsoever was observed coming from the cells. Presumably, this represents a lag between loss of calcein fluorescence from the cytosol and permeabilization of the plasma membrane, allowing entry of ethidium homodimer.

## Discussion

*Tert*-butyl hydroperoxide (tBHP) is a membrane-permeant pro-oxidant agent used in several cell lines as a model to study the effects of oxidative stress on cellular function and cell death pathways [18,19,21,22]. Once inside cells, tBHP generates *tert*-butoxy radicals inducing several physiological alterations with consequent loss of cell viability via apoptosis or necrosis [28]. The type of effect is dependent on the cell line or primary culture type, tBHP concentration, and exposure period. Lipid peroxidation [28], depletion of intracellular reduced glutathione [29], modification of protein thiols [30] and cytosolic calcium deregulation [20] are some of the most common alterations.

In cardiomyocytes, tBHP induced loss of cell shape, depletion of ATP, and formation of adenosine [21]. Other studies demonstrated that treatment with 1 mM tBHP lead to a rise in intracellular calcium concentration, hyper-contracture and loss of membrane integrity in cardiac myocytes isolated from rat ventricles [31]. To the best of our knowledge, however, morphological alterations in cardiomyocytes or myoblasts during tBHP-induced cell death has not been reported.

In the present study, tBHP was used as a model compound to characterize morphologic changes in H9c2 cells resulting from oxidative stress. It was found that 1 hour treatment with 50 μM tBHP induces an increase in the oxidation of the probe DCF, which indicates the presence of intracellular oxidative stress. The data also indicates that after 6 hours treatment with 50 and 100 μM tBHP, cell death and detachment occurred. These effects were prevented by N-acetylcysteine (NAC) and Trolox. NAC is the acetylated form of the amino acid L-cysteine and a source of sulfhydryl (SH) groups. In the body, NAC is converted into metabolites capable of stimulating glutathione (GSH) synthesis and can also act directly as a free radical scavenger, due to its nucleophilic and antioxidant properties [32,33]. Trolox is a water-soluble analogue of vitamin E lacking the phytyl chain, with strong antioxidant properties [34]. As reported in Figure 2 (top panel) both antioxidant compounds prevented the cytotoxic effect induced by tBHP, supporting the hypothesis that ROS production in H9c2 cells is responsible for the cell death that occurs following tBHP treatment. Moreover, vital imaging studies demonstrate that some features of apoptosis, including cell rounding, membrane blebbing, and chromatin condensation, occur in cells undergoing tBHP-induced oxidative stress. However, cells also remain calcein-positive, and mitochondrial membrane potential can persist for quite some time in these apoptotic cells.

Apoptosis is a cell death program that is dependent on ATP, most of which is normally produced by mitochondria under aerobic conditions. Although tBHP-treated apoptotic cells displayed significant changes in mitochondrial morphology, many altered mitochondrial remained polarized. On the other hand, most necrotic cells lacked polarized mitochondria. The results suggest that functional mitochondria are necessary for tBHP-induced apoptosis in H9c2 cells. Nevertheless, although morphologically compromised, the mitochondrial network retains sufficient function to continue producing enough ATP to drive apoptosis in oxidatively damaged cells. Transformation of filamentous mitochondria to small spherical forms, as observed in this study, has been termed the "thread-grain transition" and has been proposed to represent a mechanism to isolate a damaged part of the mitochondrial system from the rest of the mitochondrial network [35]. According to this hypothesis, thread-grain transitions represent an obligatory step in mitochondrial-mediated apoptosis. In addition to thread-grain transitions, we noted that polarized mitochondria became concentrated near the nuclear region upon treatment with tBHP. Skulachev et al. [35] proposed that small mitochondria around the nucleus may serve to more rapidly direct some apoptotic proteins to their nuclear targets. Alternatively, concentrating polarized mitochondria around the nucleus could be an important mechanism to supply the energy needed by the nucleus during the apoptotic program.

Immunocytochemistry of H9c2 cells revealed the formation of Bax aggregates near the mitochondrial network after tBHP treatment (Figure 4). This labeling appeared to be most pronounced in areas of the mitochondrial network that displayed the weakest Mitotracker Red labeling. Mitotracker Red is a widely used mitochondrial marker, which is membrane potential dependent (according to the manufacturer). In these experiments, control and tBHP-treated cultures were processed at the same time and under identical conditions for Mitotracker, Bax and Hoechst labeling. Both control and experimental samples were photographed in the same session using identical microscope and camera settings. Visual examination showed consistent differences in fluorescence intensities of these probes between control and tBHP-treated groups, and statistical analyses of fluorescence values from digitized images demonstrated a significant inverse relationship between Mitotracker Red and Bax immunolabeling (Figure 6, lower panel). We also observed a direct association between Bax immunolabeling and Hoechst staining, showing that increased Bax labeling accompanies the stronger Hoechst labeling, associated with chromatin condensation in apoptotic tBHP-treated H9c2 cells.

Bax has been suggested to be translocated from the cytosol of cardiomyocytes in two distinct phases [36]. The first phase involves the Bax-induced release of cytochrome c, and the second phase involves packaging of Bax monomers close to mitochondria. Our results are consistent with this model in that we observed a translocation of Bax around mitochondria upon treatment with tBHP, which is further evidence that tBHP is inducing apoptosis. Considering that Bax translocation to mitochondria can be accompanied by morphological alterations in these organelles, we hypothesized that the mitochondrial permeability transition (MPT) could be involved as well. It has been reported by other authors that the mitochondrial permeability transition is an oxidative stress-dependent mechanism [37]. As tBHP induces oxidative stress in H9c2 cells, we also suspected that the MPT could be another reason for tBHP-induced H9c2 cytotoxicity. In order to test this hypothesis, we examined whether tBHP cytotoxicity could be prevented with cyclosporin-A, a MPT inhibitor. The results (Figure 2, bottom panel) showed that cyclosporin-A did not prevent cell death induced by tBHP, indicating that the MPT does not appear to be cause for the cytotoxic effect induced by tBHP under our experimental conditions.

Changes in plasma membrane asymmetry are one of the earliest features of cells undergoing apoptosis. In apoptotic cells, phosphatidylserine (PS) is translocated from the inner to the outer leaflet of plasma membrane, which serves as a recognition signal for macrophages [38]. Annexin V binds PS, and is commonly used to detect externalized PS in apoptotic cells. However, because Annexin V can also label non-externalized PS in necrotic cells with compromised plasma membranes, propidium iodide (PI) is commonly used together with annexin V to identify and distinguish necrotic from apoptotic cells [39]. Our results demonstrate that tBHP-treatment is able to induce the externalization of PS, as evidenced by the presence of annexin V-positive but PI-negative cells. Additionally, treatment with lower concentrations of tBHP for short periods of time induced an increase in the number of nuclei showing condensed chromatin, characteristic of apoptosis (Figure 6).

Cell membrane behavior after treatment with tBHP was also evaluated by DIC imaging (Figures 3, 5 and 7). tBHP-treated H9c2 cells shrank in size while undergoing membrane blebbing and releasing apoptotic bodies. Nevertheless, during this period, cell membrane integrity is maintained as seen by the maintenance of calcein fluorescence inside the cells and PI exclusion (Figure 7). In many cells, this apoptotic period was followed by necrosis, and release of cellular contents.

## Conclusion

In conclusion, exposure of H9c2 cells to tBHP results in: 1) an increase in ROS production, 2) cell rounding and membrane blebbing, 3) alterations in the mitochondrial network, including peri-nuclear clustering and thread-grain transitions, 4) the appearance of immunoreactive Bax deposits around depolarized mitochondria, 5) translocation of PS to the outer leaflet of the plasma membrane, and 6) chromatin condensation and nuclear shrinkage. All of these changes are suggestive of apoptosis; however, depending on the concentration and exposure time, H9c2 cells can also undergo necrosis, as evidenced by a rapid loss of plasma membrane integrity. Our results demonstrate the causal relationship between increased oxidative stress and cell death and emphasize the importance of vital imaging of intact cells to more completely understand the morphological and functional changes that occur during the induction of different modes of cell death by toxic chemicals.

## Methods

### Reagents

Dulbecco's modified Eagle's medium (DMEM), penicillin, streptomycin, fetal bovine serum and Trypsin-EDTA were purchase from Gibco-Invitrogen (Grand Island, NY). *Tert-*butyl hydroperoxide, N-acetylcysteine, Trolox and Cyclosporin-A were obtained from Sigma (St Louis, MO). Hoechst 33342, tetramethylrhodamine methyl ester (TMRM), calcein-AM, propidium iodide, ethidium homodimer, Mitotracker Red CMXROS and 5-(and-6)-chloromethyl-2', 7'-dichlorodihydrofluorescein diacetate (CM-H_2_DCFDA) were obtained from Molecular Probes (Eugene, OR). Annexin V-FITC and mouse anti-BAX monoclonal antibody were purchased from BD Bioscience Pharmingen (San Diego, CA). Secondary antibody FITC-conjugated anti-mouse IgG antibody was purchased from Jackson ImmunoResearch Laboratories, Inc. (Cambridgeshire, UK).

### Cell culture

The H9c2 cell line was originally derived from embryonic rat heart tissue using selective serial passages [1] and was purchased from America Tissue Type Collection (Manassas, VA; catalog # CRL – 1446). Cells were cultured in DMEM medium supplemented with 1.5 g/L sodium bicarbonate, 10% fetal bovine serum, 100 U/ml of penicillin and 100 μg/ml of streptomycin in 75 cm^2 ^tissue culture flasks at 37°C in a humidified atmosphere of 5% CO_2_. Cells were fed every 2 – 3 days, and sub-cultured once they reached 70 – 80% confluence in order to prevent the loss of differentiation potential. For epifluorescence or confocal microscopy, cells were seeded at a density of 35,000 cells per ml in glass-bottom dishes (Mat-Tek Corporation, Ashland, MA). For sulforhodamine B assays, cells were seeded as described above but in 24 well plates (final volume of 1 ml/well). For detection of PS exposure, chromatin condensation studies and immunocytochemistry assays cells were seeded in six-well plates containing glass coverslips (final volume of 2 ml/well). Cells were seeded in DMEM with 10% FBS and experiments were carried out up to 6 hours post-treatment. tBHP was added directly to the cell culture media at the concentrations described.

### Detection of intracellular oxidative stress with 5-(and-6)-chloromethyl-2', 7'-dichlorodihydrofluorescein diacetate (CM-H_2_DCFDA)

H9c2 cells seeded in glass-bottom dishes were incubated with CM-H_2_DCFDA (7.5 μM) for 1 hour at 37°C in the dark. Media was then replaced by new pre-warmed DMEM and then cells were returned to the incubator for another hour. Media was then again replaced by 2 ml of Krebs buffer (1 mM CaCl_2_; 132 mM NaCl; 4 mM KCl; 1.2 mM Na_2_HPO_4_; 1.4 mM MgCl_2_; 6 mM Glucose; 10 mM HEPES, pH 7.4). Cells were observed by epifluorescence microscopy using a Nikon Eclipse TE2000U microscope (fluorescein filter) and images were obtained using Metamorph software (Universal Imaging, Downingtoen, PA).

### Cytotoxicity and cell density evaluation by sulforhodamine B (SRB) assay

H9c2 cells seeded in 24-well plates were individually pre-incubated for 2 hours with 100 μM NAC, 200 μM Trolox or 5 μM cyclosporin-A. The concentration of inhibitors used was the maximum amount that did not cause cell death by itself. Following pre-treatment, cells were incubated with or without 50 or 100 μM tBHP for 6 hours (NAC, Trolox and cyclosporin-A were maintained in the incubation media). After treatment, the incubation media were removed and cells were washed with PBS and fixed in ice cold methanol, containing 1% acetic acid for at least 30 minutes. Cells were then incubated with 0.5% (wt/vol) sulforhodamine B dissolved in 1% acetic acid for 1 hour at 37°C. Unbound dye was removed by several washes with 1% acetic acid. Dye bound to cells proteins was extracted with 10 mM Tris base solution, pH 10, and the optical density of the solution was determined at 540 nm. Results were expressed as a function of control without tBHP treatment. Controls with vehicle (ethanol or DMSO) were also performed and had no difference from control (data not shown).

### Triple labeling of H9c2 cells with TMRM, Hoechst 33258 and calcein-AM and visualization by confocal microscopy

H9c2 cells in glass-bottom dishes were incubated with TMRM (100 nM), Hoechst 33342 (1 μg) and calcein-AM (300 nM) for 30 minutes at 37°C in the dark. Due to the very low fluorescence of the probes in the extracellular media, the images were collected without replacing the cell culture media. The images were obtained using a Nikon C-1 laser scanning confocal microscope. TMRM signal was acquired using a green He-Ne laser with the gain around 100 (pixel dwell, 30 μs). The calcein-AM signal was acquired using an air-cooled argon laser with the gain equal to 55 (pixel dwell, 20 μs). The Hoechst signal was obtained by using a violet diode laser, gain used for detection being 100 (pixel dwell, 30 μs). DIC images using the confocal microscope were collected using the air-cooled argon laser and the appropriate detector.

### Immunocytochemistry

H9c2 cells, seeded on glass coverslips in 6 well plates, were incubated with or without 50 and 100 μM tBHP during 1 hour. After treatment, cells were incubated with Mitotracker red (125 nM) for 30 minutes at 37°C in the dark, washed with cold PBS and fixed with 4% paraformaldehyde during 15 min at room temperature. Cells were rinsed with PBS and stored in PBS-T (PBS supplemented with 0.05% tween-20) at 4°C until use. Fixed cells were blocked with 1% milk in PBST during 1 h at 37°C, probed with anti-BAX mouse monoclonal antibody (2 hours at 37°) and stained with FITC-conjugated anti-mouse antibody (2 hours at 37°). Cells were observed by epifluorescence microscopy using a Nikon Eclipse TE2000U microscope. The fluorescein filter was used for FITC imaging and the rhodamine filter for Mitotracker red fluorescence imaging. Images were obtained using Metamorph software (Universal Imaging, Downington, PA).

### Annexin V/Propidium Iodide assay

Annexin V-FITC (fluorescein isothiocyanate) was used in conjunction with a vital dye, Propidium Iodide (PI), to distinguish apoptotic (Annexin V-FITC positive, PI negative) from necrotic (AnnexinV-FITC positive, PI positive) cells. After treatment with tBHP, culture media was removed, cells were washed twice with ice-cold 1× PBS and incubated with 100 μl of Annexin V Incubation Reagent (10 mM Hepes, pH 7.4, 150mM NaCl, 5mM KCl, 1mM MgCl_2_, 2.5mM CaCl_2 _supplemented with 5 μg/ml PI and with Annexin V-FITC diluted 1:20) during 15 min at room temperature in the dark. After 2 washes with 1× binding buffer (10mM Hepes, pH 7.4, 150mM NaCl, 5mM KCl, 1mM MgCl_2_, 2.5mM CaCl_2_) cells were observed by fluorescence microscopy using an epifluorescence Nikon Eclipse TE2000U microscope and images were obtained using Metamorph software (Universal Imaging, Downingtown, PA).

### Chromatin condensation detection

Nuclear morphology of cells was studied by using the cell-permeable DNA dye Hoechst 33342. Cells with homogeneously stained nuclei were considered to be normal, whereas the presence of chromatin condensation in non-mitotic cells was indicative of apoptosis. After tBHP treatment, cells were washed twice with PBS, fixed with 2 ml of ice cold absolute methanol and stained with 1 μg/ml of Hoechst 33342 for 30 minutes at 37°C in the dark. Nuclear morphological changes were detected by using an epifluorescence Nikon Eclipse TE2000U microscope (UV filter). Two hundred cells from several randomly chosen fields were counted and the number of apoptotic cells was expressed as a percentage of the total number of cells.

### Apoptosis/Necrosis transition with calcein-AM and Ethidium Homodimer

H9c2 cells in glass-bottom dishes were incubated with 100 μM tBHP for 90 minutes in the absence of any fluorescence probe. After 90 minutes, cells were incubated with ethidium homodimer (EH-1, 1 μM) and calcein-AM (300 nM) for 30 minutes at 37°C in the dark. Again, due to the low fluorescence of the probes in the extracellular media, the images were collecting without replacing the cell culture media. Cell fluorescence was collected by using an epifluorescence Nikon Eclipse TE2000U microscope. The fluorescein filter was used for calcein imaging and the rhodamine filter for EH-1 fluorescence imaging. Images in DIC, calcein and EH-1 channels were collected every two minutes during 45 minutes after a total of 120 min. treatment with 100 μM tBHP. Neutral density filters were used in order to reduce photo-damaging effects. At the end of the experiment, some random fields that were not irradiated by light were observed to guarantee that the observed morphological features of apoptosis/necrosis were not due to light-induced effects (data not shown)

### Statistical analysis

Data are expressed as mean ± SEM for the number of experiments indicated in the legends of the figures. Multiple comparisons were performed using one-way analysis of variance (ANOVA) followed by a Bonferroni post-hoc test. Significance was accepted with *p value *< 0.05.

## Authors' contributions

VS carried out the Hoechst, Annexin V, CM-H_2_DCFDA and BAX labeling, participated in epifluorescence microscopy imaging and drafted the manuscript. PO and JH carried out confocal microscopy imaging and mitochondrial labeling. JH, CO and KW participated in the design and coordination of the study and helped to revise the final version of the manuscript. All authors read and approved the final manuscript.
